# High-frequency ultrasound detection of cell death: Spectral differentiation of different forms of cell death *in vitro*

**DOI:** 10.18632/oncoscience.319

**Published:** 2016-09-12

**Authors:** Maurice M. Pasternak, Ali Sadeghi-Naini, Shawn M. Ranieri, Anoja Giles, Michael L. Oelze, Michael C. Kolios, Gregory J. Czarnota

**Affiliations:** ^1^ Department of Radiation Oncology, Sunnybrook Health Sciences Center, Toronto, ON, Canada; ^2^ Department of Laboratory Medicine & Pathobiology, University of Toronto, ON, Canada; ^3^ Physical Sciences, Sunnybrook Research Institute, Toronto, ON, Canada; ^4^ Department of Medical Biophysics, University of Toronto, Toronto, ON, Canada; ^5^ Department of Radiation Oncology, University of Toronto, Toronto, ON, Canada; ^6^ Department of Electrical and Computer Engineering, University of Illinois, IL, U.S.A.; ^7^ Department of Physics, Ryerson University, Toronto, ON, Canada

**Keywords:** quantitative ultrasound, apoptosis, oncosis, imaging, midband fit

## Abstract

High frequency quantitative ultrasound techniques were investigated to characterize different forms of cell death *in vitro*. Suspension-grown acute myeloid leukemia cells were treated to cause apoptosis, oncosis, mitotic arrest, and heat-induced death. Samples were scanned with 20 and 40 MHz ultrasound and assessed histologically in terms of cellular structure. Frequency-domain analysis of 20 MHz ultrasound data demonstrated midband fit changes of 6.0 ± 0.7 dBr, 6.2 ± 1.8 dBr, 4.0 ± 1.0 dBr and −4.6 ± 1.7 dBr after 48-hour cisplatinum-induced apoptosis, 48-hour oncotic decay, 36-hour colchicine-induced mitotic arrest, and heat treatment compared to control, respectively. Trends from 40 MHz ultrasound were similar. Spectral slope changes obtained from 40 MHz ultrasound data were reflective of alterations in cell and nucleus size. Chromatin pyknosis or lysis trends suggested that the density of nuclear material may be responsible for observed changes in ultrasound backscatter. Flow cytometry analysis confirmed the modes of cell death and supported midband fit trends in ultrasound data. Scatterer-size and concentration estimates obtained from a fluid-filled sphere form factor model further corresponded with spectral analysis and histology. Results indicate quantitative ultrasound spectral analysis may be used for probing anti-cancer response and distinguishing various modes of cell death *in vitro*.

## INTRODUCTION

Cell death introduces significant alterations in physical and morphological characteristics of cells and nuclei [1]. It can be categorized into different forms including apoptosis (programmed cell death), oncosis, mitotic arrest (death in mitosis), and coagulative cell death (e.g., due to heating). These forms share many common features, to the degree that they can be often misclassified during assessment of biological samples. Even at a molecular level, recent studies have indicated that markers such as TUNEL positivity [2, 3], or the Annexin V positive / propidium iodide negative phenotypes [4, 5] are not unique to apoptosis. However, these various forms of cell death have distinct morphological features which are associated with potentially-unique viscoelastic properties that alter their acoustic properties and ultrasound scattering. These differences may potentially be used for the identification and discrimination of different forms of cell death. Notably, necrosis should not be considered a form of cell death, but rather as the end stage to any cell death process, as outlined by Majno and Joris [1].

Determining the dominant form of cell death present during therapy is critical in assessing treatment efficacy and in establishing appropriate treatment dosages, as varying concentrations of the same drug can lead to different outcomes [6, 7, 8]. If the dominant mechanism involves apoptosis, a highly-controlled process involving the sequestration of aggregated chromatin into subcellular bodies [9] that become phagocytosed by macrophages [10], then minimal inflammation is induced. In contrast, cells that die by oncosis have dysregulated membrane ion pumps [1, 11], leading to the release of pro-inflammatory molecules into nearby tissue when these cells burst from hydrostatic pressure [12]. Due to the difficulty in establishing appropriate molecular markers for discriminant identification of cell death forms, assessments on the basis of morphological criteria are currently considered more reliable [13, 14]. However, most morphological evaluation of tissue samples requires invasive biopsy to collect samples for histology.

Ultrasound is an inexpensive, portable, and rapid imaging modality that is frequently used in clinic for cancer imaging [15, 16]. Ultrasound imaging of tumors relies on the fact that the physical properties of tissues (i.e. density, stiffness, scatterer distribution, shape, and diameter) change during the course of disease progression or in response to anti-cancer therapies, which translates to altered acoustic scattering [17, 18]. High frequency ultrasound (HFUS) extends this concept to frequencies of 20 MHz and higher to offer greater sensitivity in detecting physical and acoustic alterations on the cellular and sub-cellular level. At this frequency range, quantitative parameters derived from spectral analysis of ultrasound radiofrequency (RF) data (raw ultrasound data) have been shown to be sensitive to changes in cell structure of treated cancer cells *in vitro* and *in vivo* [19].

It has been demonstrated previously that the frequency-dependent data contained within the HFUS RF signal can be reflective of structural changes in tissue at the cellular level [17, 20, 21, 22]. Specifically, spectral parameters such as mid-band fit (MBF), 0-MHz intercept, and spectral slope can be extracted from ultrasound RF data using linear regression analysis of normalized backscatter power spectrum [23, 24, 25]. It was previously demonstrated that these parameters are linked to the size and concentration of effective scatterers, and the relative impedance difference between the effective scatterers versus surrounding media [20, 21, 22]. These spectral parameters have been demonstrated to be effective in characterizing normal and tumor cells in addition to differentiating viable from apoptotic cancerous tissue [19, 26, 27, 28]. In particular, previous studies revealed a 16-fold increase in the backscatter intensity for apoptotic cells relative to viable cells along with significant increases in other parameters such as the spectral slope [19, 26].

Other quantitative ultrasound spectral analysis techniques have also been proposed based on fitting form-factor models to frequency-dependent backscatter coefficients in order to estimate bioacoustic properties of tissues [29, 30, 31]. The backscatter parameters derived using these techniques include the average acoustic concentration (AAC) and average scatterer diameter (ASD), which provide additional evidence to support the changes observed in the spectral parameters during cell death processes [32]. The AAC is defined as the product of the number of scatterers per unit volume (density) and the squared difference in acoustic impedance of effective scatterers versus the surrounding medium [33]. The backscatter parameters have also previously been demonstrated to be capable of being used to differentiate between benign versus malignant tissues *in vivo* in animal tumor models [29].

The investigation here seeks to expand on previous work beyond the characterization of apoptotic versus viable cells by investigating the efficacy of high-frequency quantitative ultrasound techniques to discriminate between different forms of cell death. Scatterer-size estimates, and acoustic concentration estimates are used in this study to addresses the capacity of HFUS to discern different phases of each form of cell death relative to the initial viable state. The modes of cell death studied include classic p-53-dependent apoptosis induced by cisplatinum, serum deprivation-induced oncosis, colchicine-induced mitotic arrest, and heat death by immersion in a hot bath for an extended period of time. Quantitative ultrasound spectral and backscatter parameters were derived from ultrasound RF data acquired prior to and at different times after treatment. In parallel, flow cytometry analysis was performed to correlate light scattering (forward versus side scattering after gating for non-viable populations) with observations from ultrasound scattering for the different modes of cell death induced. Histological analysis assessed changes in chromatin content, as chromatin is hypothesized to be a major scatterer of ultrasound in tumor cell populations undergoing cell death. Results indicated that acoustic parameters such as midband fit were found to be capable of differentiating forms of cell death in correlation with side light scatter trends and histology, indicating that the structural status of chromatin is responsible for these observations. These findings suggest that quantitative ultrasound spectral analysis may be a viable option for probing anti-cancer response under various forms of death and distinguishing these forms from one another *in vitro*.

## RESULTS

### Ultrasound data and cell structure

**Figure 1:**
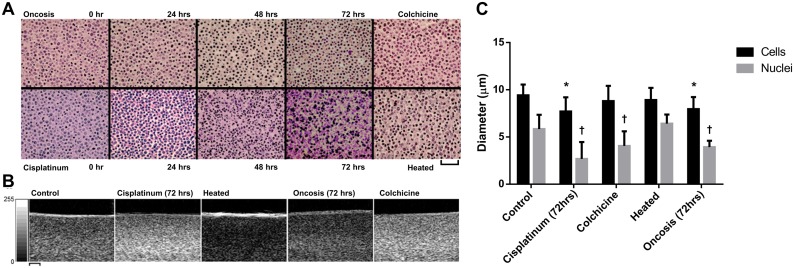
**A.** Representative histology shown from individual and time-course experiments. From left to right, apoptotic and oncotic cells are observed in the cisplatinum and decay treatments, respectively. Note the disparity in cell organization and distribution of potential scatterers between the two mechanisms, with apoptosis displaying substantially greater arrangement of condensed chromatin at 72 hours. Heat death and colchicine treatments are shown on the right-most panels, where sudden death and mitotic arrest are displayed, respectively. **B.** Representative B-mode ultrasound scans at 20 MHz display the effects of morphological and structural changes to samples on the backscatter intensity. Notably, all cell forms of death involving condensation of DNA were associated with increases in B-mode speckle intensity (intensity scale bar). **C.** Cell and nuclei average diameter measurements for 72-hour cisplatinum, 36-hour colchicine, heat death, and 72-hour oncotic decay treatments. These data represent a mean of 40 measurements taken from 2 independent H&E stained slices per sample. Error bars represent SD. * indicates p<0.01 for whole cell measurements relative to the control. † indicates p<0.01 for nuclei measurements relative to the control nuclei.

B-mode images (Figure 1) displayed significant alterations in speckle intensity as a result of all treatments. Histology indicated changes in cell structure presented in Figure 1 and described further below. Data obtained at treatment times with cells containing condensed and non-lysed chromatin (cisplatinum at 48 hours and colchicine at 36 hours) generally featured increases in speckle intensity, consistent with a greater degree of ultrasound backscatter intensity from samples. Comparatively, treatments involving the lysis or denaturation of DNA (oncotic decay at 72 hours and heat treatment, respectively) had noticeably lower speckle intensity.

**Figure 2:**
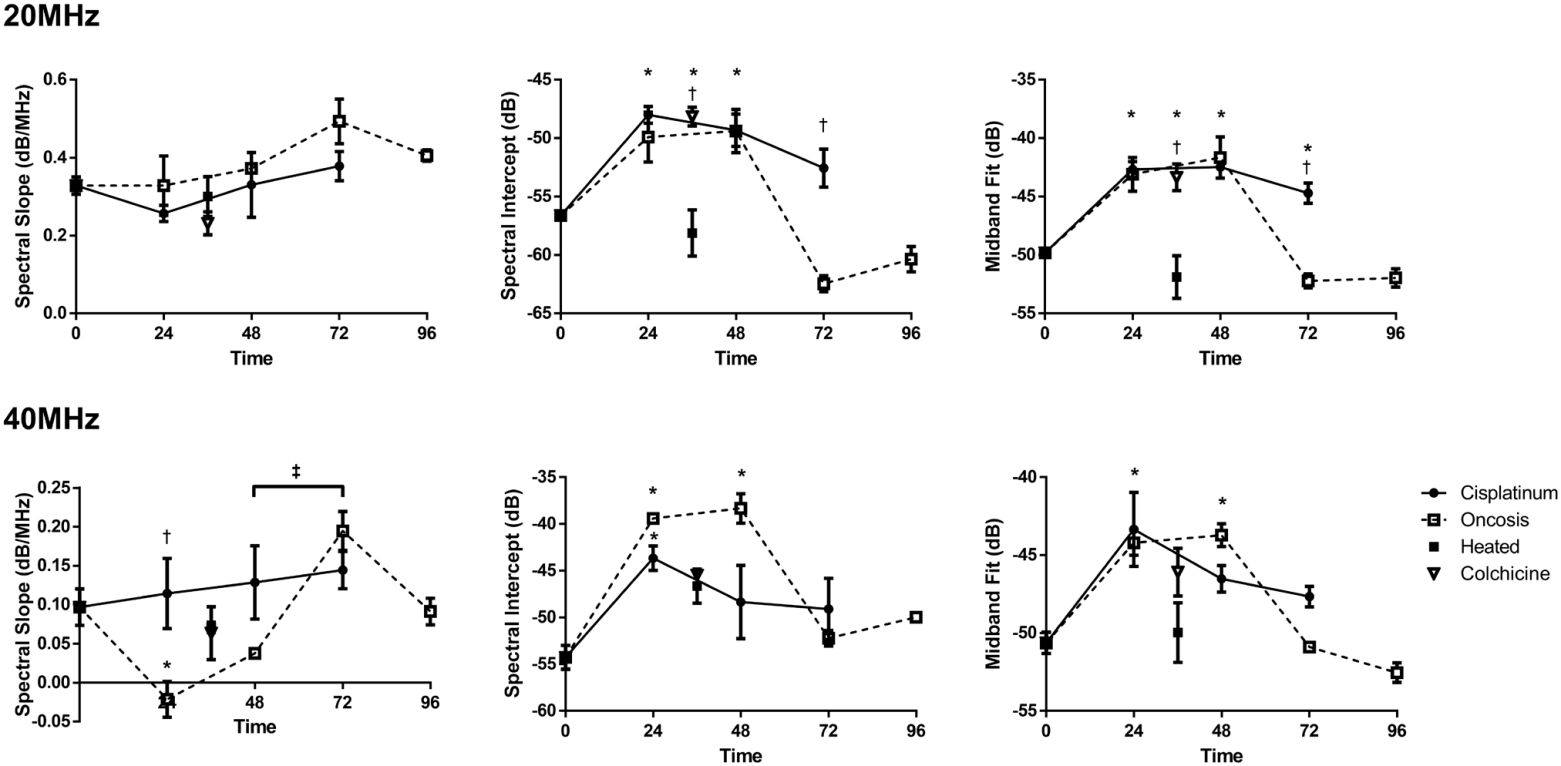
High-frequency ultrasound spectral parameters for 20 MHz (top row) and 40 MHz (bottom row) center frequencies for time-course treatments Error bars represent SE at n=3. * indicates p<0.05 significant differences between the indicated time-point for one or more forms of cell death relative to the control. † indicates p<0.05 significant differences between one or more forms of cell death at the indicated time point. ‡ indicates p<0.05 significant difference between indicated time points for oncotic cell death only.

Quantitative analysis of the RF signal was found to be effective in differentiating forms of cell death at experimental times and results were co-incident with observed morphological alterations from histology. As previously reported, the apoptotic response due to cytotoxic cisplatinum in AML cells was clearly evident in changes of the midband fit (MBF) parameter with data obtained from both the 20 MHz and 40 MHz center frequency transducers (Figure 2). The MBF parameter at 20 MHz increased from −49.8 ± 0.6 dBr to −42.7 ± 1.2 dBr (p<0.001) as early as 24 hours after exposure to cisplatinum in order to induce apoptosis, and remained at elevated levels for further times. Both colchicine-induced mitotic arrest and ischemia-induced oncosis followed a similar increase in MBF at 20 MHz. Here the MBF increased to −43.4 ± 2.0 and dBr −43.1 ± 2.5 dBr for mitotic arrest and ischemia-induced oncosis, respectively. In contrast to apoptosis, samples undergoing oncosis eventually exhibited decreased MBF values by 72 hours back down towards levels of the control. Samples of cells killed through heat treatment did not display any significant increase in MBF. In general, the trends in these results were recapitulated at the 40 MHz frequency, with the exception that the MBF values dropped down to statistically-similar values to the control at the earlier 48-hour time point. For all frequencies, changes in 0-MHz intercept were similar to those of the MBF (Figure 2).

At 20 MHz, the spectral slope was found to be statistically similar between all forms of cell death and at each experimental time per cell death form relative to the control (Figure 2). However, significant changes occurred in this parameter at the higher resolution 40 MHz frequency. An increasing trend was observed for cisplatinum-induced apoptosis over the course of 72 hours. Samples undergoing oncosis revealed three phases of spectral slope change. Within the first 24 hours there was a decrease from 0.10 ± 0.05 dBr/MHz to −0.02 ± 0.04 dBr/MHz (p<0.05 compared to control) followed by an increase to 0.19 ± 0.03 dBr/MHz (p<0.01 compared to 24-hour oncosis) by 72 hours before a final decrease to 0.09 ± 0.02 dBr/MHz, a value that is statistically similar to the control. In addition, at this frequency neither colchicine treatment nor heat-induced cell death resulted in any significant changes to the spectral slope.

Overall, spectral parameters were observed to be sensitive in discriminating forms of cell death at times after cell death induction. Notably, the 40 MHz spectral slope was shown to be the earliest discriminator between apoptosis and oncosis, with significant differences observed as early as 24 hours. At later times, the MBF and 0-MHz intercept parameters for the 20 MHz transducer served as a viable means for differentiating forms of cell death. Further discriminatory power was attained through the use of form-factor model scatterer estimates (below).

### Scatterer estimates

**Figure 3:**
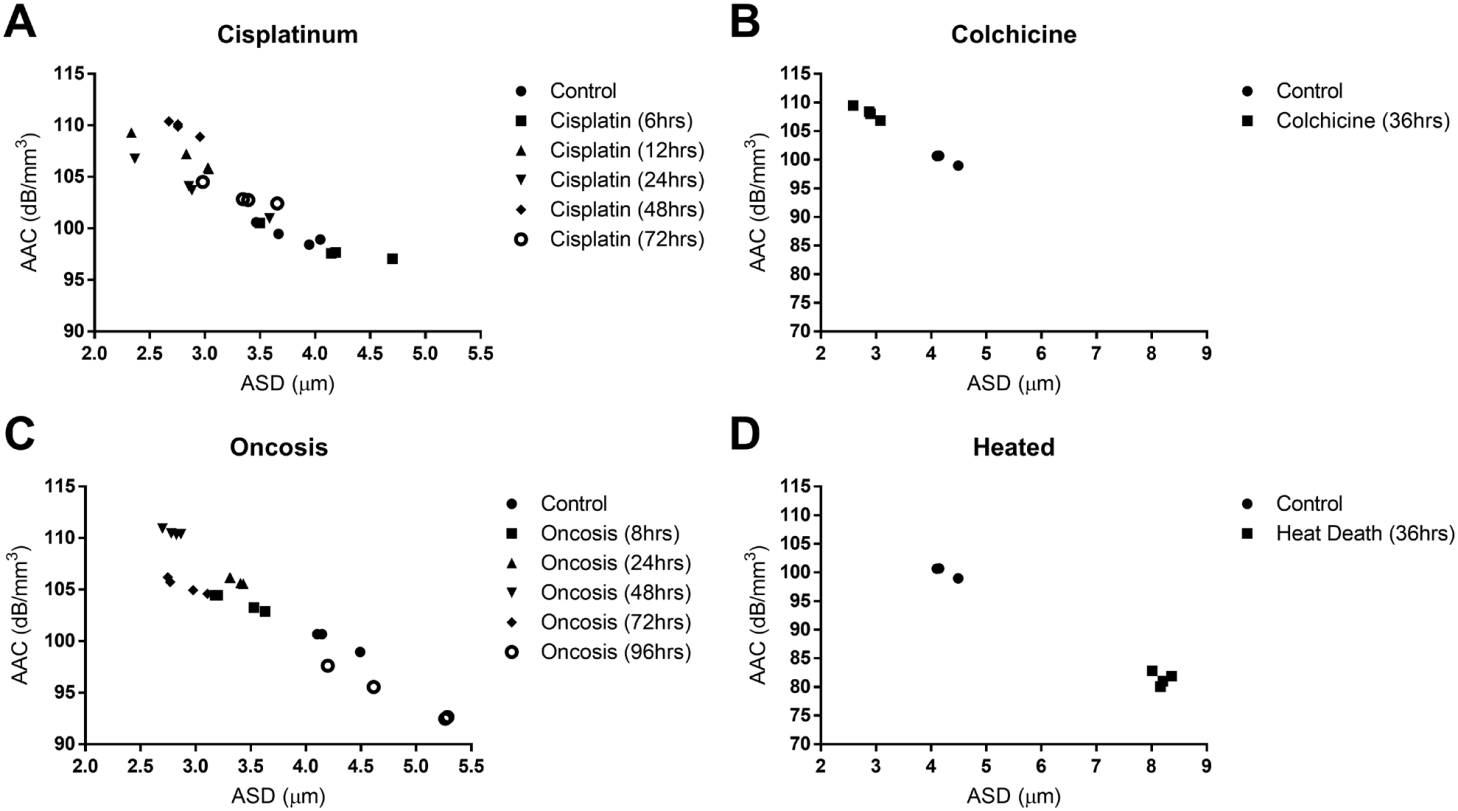
Estimates calculated from 40 MHz RF data with the fluid filled sphere scattering model are shown for the treatments: A. cisplatinum, B. colchicine, C. oncotic decay, and D. heat treatment This data was plotted as scatterplots displaying average acoustic concentration (AAC) on the vertical axis against average scatterer diameter (ASD) on the horizontal axis. At 40 MHz, these data represent nuclei and fragments of nuclei as the primary scatterers. Data indicates that treatments known to induce DNA condensation cause the formation of multiple, small scatterers at early time points (≤ 48 hours).

It was observed that the primary scatterers in the cell samples were more accurately described by the Anderson Fluid-filled sphere form-factor model at 40 MHz (Figure 3) compared to lower resolution data obtained at 20 MHz. For all samples considering AAC versus ASD, there was a very strong correlation between the two parameters at 40 MHz (Pearson r value = −0.96), which indicated effective scatterer size and effective scatterer concentration were both changing (Figure 3). Data collected at 20 MHz did not yield similar results and displayed a weaker correlation (Pearson r value = −0.59). For 40 MHz data, effective acoustic scatterer concentration estimates indicated an increase from 100.0 ± 3.5 dBr/mm^3^ to 109.8 ± 0.7 dBr/mm^3^ and 110.5 ± 0.3 dBr/mm^3^ by 48 hours for cisplatinum-induced apoptosis and decay oncosis, respectively. The corresponding scatterer diameter decreased from 3.9 ± 0.4 μm, to 2.9 ± 0.5 μm and 2.8 ± 0.1 μm for cisplatinum-induced apoptosis and oncotic decay after 48 hours, respectively. For heated samples, the ASD increased to 8.2 ± 0.1 μm and the AAC decreased to 81.4 ± 1.2 dBr/mm^3^, whereas colchicine treated samples demonstrated an ASD decrease to 2.9 ± 0.2 μm and an AAC increase to 108.2 ±1.1 dBr/mm^3^. These findings were in good agreement with overall changes in the spectral slope and MBF parameters for all treatments (24-96 hours inclusive). Nevertheless, the scatter estimates were sensitive to cell changes earlier than 24 hours, with AAC increases to 107.1 ± 1.6 dBr/mm^3^ and 105.9 ± 0.3 dBr/mm^3^ for 12-hour cisplatinum treatment and 8-hour oncotic decay, respectively (Figure 3).

### Histology

Gross morphological alterations in samples were observed in the course of treatments and corresponded well to changes in ultrasound data as described above (Figure 1A and 1B). Prior to treatment, cells demonstrated a uniform staining of nuclear material. Colchicine-treated samples demonstrated increased amounts of mitotic cell staining due to duplicated DNA. Cisplatinum-induced apoptosis, ischemic oncosis, and colchicine-induced mitotic arrest all demonstrated significant condensation of nuclear material associated with cell death. Cisplatinum-treated cells featured nuclei that decreased in diameter from the initial 5.8 ± 1.5 μm to 2.7 ± 1.8 and cells that decreased in diameter from 9.4 ± 1.1 μm to 7.7 ± 1.5 μm by 48 hours (Figure 1C). Significant karyorrhexis and morphological changes were observed following 72 hours of cisplatinum treatment. Apoptotic nuclear material breakdown and cellular blebbing were evident in the cisplatinum treated samples. Clonogenic assays (Figure Supplementary S1) indicated that no cisplatinum-treated cells were viable by this experimental time.

In contrast, oncotic cells displayed a greater degree of cells with nuclear lysis, with both cells and nuclei having decreased in size by the 72-hour time point from control 9.4 ± 1.1 μm and 5.8 ± 1.5 μm, to 8.0 ± 1.3 μm and 3.9 ± 0.7 μm for cells and nuclei, respectively (Figure 1C). Clonogenic assays confirmed less than 0.1% viable cells after 48 hours of serum deprivation. Colchicine treatment resulted in an increased proportion of cells with condensed chromatin and no signs of karyorrhexis or karyolysis. Measurements revealed an expected decrease in nuclear diameter as chromatin condensed from 5.8 ± 1.5 μm to 4.1 ± 1.6 μm, but no change in overall cell size (Figure 1C). Supplementary clonogenic assays demonstrated that less than 0.1% of cells remained able to divide, confirming that the vast majority of the cell population was arrested (Figure Supplementary S1). No significant gross differences in cell or nuclear size were observed for heat-treated samples. Clonogenic assays confirmed that all cells were non-viable.

### Flow cytometry

**Figure 4:**
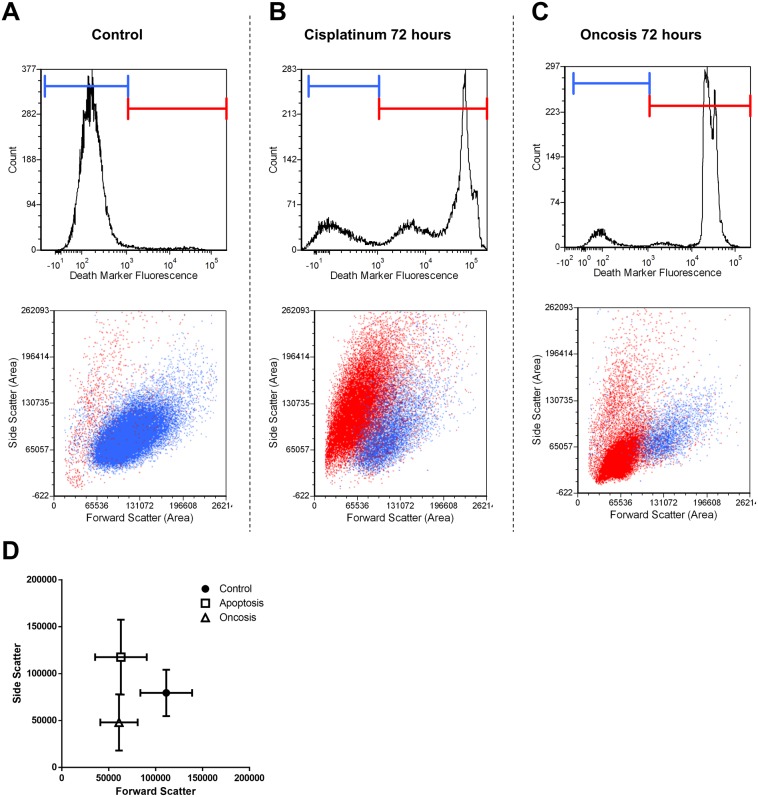
Flow cytometric differentiation of viable, apoptotic, and oncotic cells Following debris and doublet exclusion, cells were gated on the fluorescence viability indicator (propidium iodide), then backgated onto a colour dot plot, with viable cells represented as blue and non-viable represented as red. **A.** Untreated control samples generally contained viable cells which were characterized by relatively highly FSC and SSC. **B.** Cisplatinum treatment for 72 hours was followed by the appearance of an apoptotic-necrotic population featuring decreased FSC and slightly elevated SSC. **C.** Ischemic decay for 72 hours produced an oncotic-necrotic population featuring the vast majority of non-viable cells as having decreased both FSC and SSC. **D.** Scatterplot of average values for viable, apoptotic-necrotic, and oncotic-necrotic populations. All populations are visibility and statistically differentiable (p<0.01). Error bars represent SD for n=4 sample size.

Cell cycle analysis confirmed the increased percentage of cells with G2/M phase DNA content due to colchicine addition, from 8.7±2.5% in control samples to 29.7±6.1% (p<0.001) (Figure Supplementary S2). This was consistent with the known mechanism of mitotic arrest. Cisplatinum-treated samples and oncotic decay samples did not display any increase in percent G2/M phase cells, although small increases in S-phase were observed. Backgating cells based on viability marker fluorescence onto a color dot plot of forward light scatter (FSC) versus side light scatter (SSC) confirmed a visible distinction between viable, apoptotic necrotic cells, and oncotic necrotic cells at 72 hours post-treatment (Figure 4). Relative to viable cells, apoptotic necrotic cells maintained high SSC while decreasing in FSC, indicating decreases in cell size, while maintaining high intracellular scattering of light, as confirmed by histology. Oncotic necrotic cells demonstrated significant decreases in both FSC and SSC at 72 hours, indicating both a decrease in size and intracellular complexity.

## DISCUSSION

Quantitative ultrasound spectral and backscatter parameters including the spectral slope, the MBF, the 0-MHz intercept, the AAC, and the ASD have been previously used to characterize biological tissues [26, 34, 35, 36, 37, 38]. However, most of these studies have focused on one or two forms of cell death, most commonly apoptosis, which is not the only form of cancer cell death induced by anti-tumor treatment. This investigation therefore expanded the use of HFUS to compare and characterize other modes, including oncosis, mitotic arrest, and heat death in order to demonstrate its capacity for differentiating modes of cell death. The work has demonstrated that HFUS possesses a capacity to detect and discriminate between forms as early as 24 hours.

Changes in mid-band fit were readily apparent. In samples with nuclear pyknosis and karyorrhexis, the midband fit increased by almost by 10 dBr. This was consistent with previous work which associated such changes with increases in ultrasound backscatter and the general aggregation of nuclear material associated with cell death. The drop in backscatter at 48 and 72 hours of cisplatinum treatment was consistent with other work which has demonstrated that ultrasound backscatter decreases in late-stage apoptosis when nuclear material is almost completely digested. The 0-MHz parameter, which can be related mathematically to the concentration of acoustic scatterers, mirrored the changes in mid-band fit. This was consistent with the process of nuclear pyknosis forming a greater number of dense nuclear bodies serving as potential acoustic scatterers.

Changes in spectral slope also occurred with exposure of samples to cell death induction. A significant body of work has indicated that the spectral slope is related to the size of scattering structures in tissue [20, 34]. As reported previously, the spectral slope can increase in cell samples that exhibit a significant amount of apoptosis [26], indicating that the scatterers are decreasing in overall size. This was observed at 40 MHz, with an increasing spectral slope as a function of time and extensive cell death. This observation is in agreement with current understanding of the apoptotic mechanism and with flow cytometry measurements of decreasing forward light scatter (FSC) in this study. FSC decreases in apoptotic populations are often reported and correlated to cell shrinkage [39, 40]. With this mode of cell death there is observable pyknosis and karyorrhexis of the cellular DNA into macroscopic fragments [41] that collect along a degrading nuclear membrane [42]. Effectively, in terms of a working hypothesis, this turns the nucleus from a single relatively-large scatterer into multiple smaller highly-condensed scatterers. The process continues with cell membrane blebbing and the eventual compartmentalization of degraded organelles and observable fragments of condensed DNA into vesicles [9] sometimes referred to as “apoptotic bodies” [42]. This transforms the cell as a whole from a single large scattering structure into several smaller and denser structures. Histological analysis of cisplatinum-treated cell and nucleus sizes support this observation, as both cells and their nuclei had significantly smaller diameters by as early as 24 hours. Measurements of ASD and AAC were consistent with this interpretation, with increases in AAC and decreases with ASD when cells exhibited increases in nuclear bodies associated with cell death.

Serum deprivation-induced oncosis is quite different in terms of cell size alterations during the death process as a result of the failure of ionic pumps lacking energy input to maintain ion gradients. The de-energization of the Na^+^K^+^-ATPase pumps and other related pumps at membranes results in the increase of intracellular [Na^+^] and [Cl^−^], accompanied by the influx of water, resulting in the characteristic cell and organelle swelling [11]. This could reasonably explain the observed decrease in spectral slope within the first 24 hours, indicating an increase in scatterer size. Other studies have also noted a significant decrease in spectral slope at early times for cells undergoing oncosis [27, 43]. This increase in cell size is temporary, as several studies have noted that oncotic cells burst under hydrostatic pressure and release their intracellular content, often resulting in an inflammatory reaction under *in vivo* conditions [10]. This phenomenon may account for the subsequent increase in spectral slope between 24 hours and 72 hours, as a greater amount of cells shrink following cellular lysis and the release of intracellular material. Histology in this study supported this observation, as both whole cells and nuclei were determined to be reduced in diameter by the 72-hour time. Flow cytometry further validated this observation, as cells undergoing oncosis were found to significantly decrease in FSC, which is considered to result from drastic loss of cellular content and volume [4, 44]. It is also supported by the observation that serum starvation leads to unrecoverable cell death after 24 hours, by which time it is presumed that burst cells could not reverse the death process [45]. AAC and ASD measures could be used to differentiate time-dependent changes with serum deprivation. Increases in ASD and decreases in AAC over time were consistent with observations of cellular swelling and uncontrolled enzymatic lysis of nuclear material, respectively. The initial size change at early time points in oncosis is crucial to HFUS's capacity to discriminate oncosis from apoptosis, as it takes advantage of the differing trends in scatterer size changes.

As expected, there were discrepancies between data obtained at 20 MHz and at 40 MHz due to the differences in interrogating wavelengths that interact with potential scatterers. Wavelengths of 38 μm at the 40 MHz frequency would be more sensitive to cellular size changes and changes in cellular nuclear material. The changes at 20 MHz were less sensitive due to the lower resolution of the center frequency associated with the corresponding transducer. The difference between 20MHz and 40MHz ASD values is also related to the fact that the two different ultrasound frequencies are primarily interrogating different cellular structures. Studies by Oelze *et al*. [31] have demonstrated that using different frequency bands during analysis may give rise to distinct results. In this study the disparity in values may stem from the using analysis bandwidths of 10.3 MHz to 25.8 MHz and 22.1 MHz to MHz for the 20 MHz and 40 MHz measurements, respectively.

Several observations within this study have given support to the previously-stated working hypothesis that a cell's nuclear material is an effective ultrasound scatterer [46, 47]. Previous investigations noting the condensation of DNA after chemotherapeutic treatment had observed significant increases in the MBF parameter [26]. These findings were recapitulated in this study for samples undergoing cisplatinum-induced apoptosis, a mode of cell death that frequently features pyknosis and karyorrhexis [1, 42]. These processes are believed to play a key role in the observed increase in the MBF. During both apoptosis and mitosis, the increased compaction of nuclear material causes an increase in density and compressibility of scattering structure [48], both of which have a significant relationship with the acoustic impedance. By extension, the MBF is related to the relative difference in acoustic impedance between scatterers and their surrounding environment [20]. There are several lines of evidence from previous investigations, summarized in Banihashemi *et al.* [49] to support the theory that chromatin structure and organization affect ultrasound scattering, beginning with the observation of increased scattering from pyknotic nuclei found within hypoxic environments inside tumor spheroids [46, 50]. Moreover, all studies that implemented DNase enzymatic digestion of condensed nuclear material lead to a normalization of backscatter [19, 26]. Within the study here, histology demonstrated that mitotic arrest also featured the condensation of nuclear material alongside an increase in the MBF and in the overall brightness of B-mode speckle, an approximate indication of the amount of backscatter coming from the sample. Similar observations were made for oncotic cells, with other studies increasingly observing that pyknosis and karyorrhexis are also present during oncosis within similar timeframes [51, 52, 53]. Within the first 24 hours, the MBF increased for oncotic cell samples, expanding on other studies which had analyzed oncosis at shorter time points and had also observed increases in the MBF [27]. Further support for the hypothesis that DNA condensation influences ultrasound scattering comes from the observation that sudden heat death did not result in any increases to ultrasound backscatter. The temperature used in this study to induce heat death is sufficient to cause thermal denaturation of chromatin [54], leading to a non-condensed state. In this mode of cell death, the MBF did not increase, indicating less backscatter coming from heated samples.

Theoretically, scattering strength is influenced not only by changes in the mechanical properties of scatterers (i.e. chromatin condensation increasing density), but also in the number of scatterers per unit volume and, when considering the wavelengths used in this study, the distribution of scatterers [37, 55]. It is also important to note that at the interrogating wavelengths of approximately 38 μm and 76 μm for 40 MHz and 20 MHz center frequencies, respectively, and the size of AML cells (9-11 μm) and nuclei (4-6 μm) measured within this study, the realm of Mie scattering is approached. This introduces ensemble effects that complicate scattering strength analysis. AAC and ASD measurements suggest that apoptosis, mitotic arrest, and oncosis are all marked by initial increases in scatterer concentration and a smaller size of scatterers between 0 and 48 hours, which may explain the observed increase in MBF and intercept parameters. Corresponding histology also demonstrates the formation of multiple nucleic bodies that may each serve as scatterers, in agreement with literature observations of what occurs during these forms of cell death [51, 52, 53]. Histology from heat-treated samples indicates a lack of dense scatterer formation, coinciding with the observation that there is no increase in AAC or MBF.

The influence of multiple dense chromatin bodies on ultrasound scattering could also explain the differences seen in the MBF by the 72-hour time point, as apoptosis and oncosis end in two distinct necrotic states. In the absence of phagocytes, apoptotic cells reorganize into smaller compartmentalized bodies containing fragmented nuclei and patches of chromatin that remain condensed [56, 57]. These remains of condensed nuclear material may account for MBF levels remaining elevated even after 72 hours. Oncosis features the release of cellular content [58] and the uncontrolled action of DNase enzymes leading to karyolysis [59], which would have a twofold effect: a reduction in the density of chromatin bodies, as observed in histology, and a reduction in the overall number of scatters per volume. In theory, both effects would decrease Mie scattering and therefore contribute to the decrease in MBF for samples dying by oncosis at 72 hours. Again, this would be in agreement with previous studies utilizing DNase to decrease MBF to that of viable cells after chemotherapeutic treatment [19, 26]. Upon inspection of the histology images at 72 hours, the difference between cells having undergone apoptosis versus oncosis tissue is very evident. While fragmented nuclei from the cisplatinum-treated samples remained condensed, the oncotic-necrotic nuclei showed substantial structural degradation.

Flow cytometry analysis of light scattering supports these observations, as apoptotic cells retain condensed chromatin, which enhances light reflection and refraction, observed as unchanged or increased SSC [44]. In contrast, oncotic cells within this study decreased in both FSC and SSC, which current literature suggests is due to an efflux of cytosol and intracellular components that would otherwise serve as scatterers [44]. These observations have been previously reported for other cells lines in studies comparing flow cytometric profiles of apoptosis versus oncosis [4]. Considering the dependence of the MBF and SSC to the concentration and density of small scatterers of sound and light, respectively, these findings support the hypothesis that the loss of condensed chromatin content in oncosis is a significant morphological event that results in the decrease of the MBF for oncotic necrosis, but not apoptotic necrosis.

In conclusion, high-frequency quantitative ultrasound methods can be used to characterize apoptosis, oncosis, mitotic arrest, and heat-death responses in cell samples. Experimental evidence gathered within this work has added to the working hypothesis that subcellular-level changes to the cells nuclear material has a profound influence on ultrasound scattering. The results also indicate the potential of this technology in identifying the preliminary mode of cell death cancer cells undergo during anti-cancer treatment, therefore permitting the appropriate modifications to be made to maximize treatment efficacy and minimize collateral damage to a patient's body. The work lays a framework for future investigations aimed at extending these studies to *in vivo* systems and expanding the investigation to include other forms of cell death outcome such as mitotic catastrophe and autophagy.

## MATERIALS AND METHODS

### Cell culture and treatment

Acute Myeloid Leukemia (AML-5) cell line was used in this study to characterize different forms of cell death using quantitative ultrasound, histology, and flow cytometry. The AML-5 cell line was selected because its facile growth and ease of experimental manipulation could readily provide adequate bulk quantities of packed cells. In addition, this cell line has been previously shown to remain unaffected by the sample preparation procedure (i.e. centrifugation, mechanical interaction, etc.) [60]. The use of such cells was selected as a crude approximation of cell-dense tumors with minimal vasculature. We have previously investigated the apoptotic death response of this cell line using quantitative ultrasound validated by histology [61].

AML-5 cells were grown in suspension in 150 mL T-flasks. An alpha minimal essential medium (AMEM) supplemented with 5% fetal bovine serum and 1% penicillin/streptomycin was used for culturing. The AML cells were grown to a concentration of approximately 10^6^ cells/mL. In incubators at 37°C with 5% CO_2_, sets of six flasks were maintained at this concentration at 150 mL volume for the duration of all the treatments done in suspension. The treatments were as follows: cisplatinum to induce apoptosis, serum deprivation to cause oncosis, colchicine to induce mitotic arrest, and immersing samples in hot water to bring about heat death and denaturation. At least one untreated control sample (n=5) was measured per experiment alongside treated samples.

For the cisplatinum treatments (n=3), sets of flasks were exposed to cisplatinum (Sigma) at 10 μg/mL. Cisplatinum causes a p-53-dependent apoptosis in this cell line. Cells were kept for 24, 48 and 72 hours of exposure to the drug. Cells were viewed in suspension using light microscopy to confirm that apoptotic morphological changes were occurring (visually evident after 24 hours of exposure).

Other flasks of cells were treated with colchicine (n=3) at a concentration of 0.1 μg/mL for 24 hours while in suspension media. Colchicine arrested the cells at the metaphase of mitosis by inhibiting microtubule formation [62, 63] with a near 50% nominal concentration of cells presenting with mitotic bodies present in histology.

The cells were extracted from suspension by centrifugation. Treated and untreated samples were spun for 10 minutes at 500 g in a fixed angle centrifuge in 500 mL bottles. The supernatant was decanted and the condensed cells were re-suspended using phosphate-buffered saline (PBS) with calcium and magnesium supplement. The re-suspended cell samples were poured into separate 50 mL conical tubes and centrifuged again at 1000 g in a swinging bucket centrifuge for 10 minutes. After a final re-suspension in PBS, the cells were deposited into stainless steel wells and spun for 10 minutes at 2000 g to form the final condensed cell sample, mimicking the close packing of cells seen in solid malignancies. Previous studies with this cell line at these centrifuge speeds indicated that no histological differences or differences in cell packing arise due to ultrasound sample preparation [60].

Normal cells prepared in centrifuged samples as described above were treated with high heat to induce protein denaturation and coagulative cell death (n=3). Prior to scanning, centrifuged cells were placed into a water bath at 60°C and left for approximately 15 minutes. The cells were then scanned immediately afterward (described further below).

For the decay experiment to induce oncosis (n=3), centrifuged cells were kept in PBS at room temperature for 24, 48, 72 and 96-hour time courses.

### Histology

Immediately after ultrasound imaging, cell samples were fixed in 10% (w/v) formalin phosphate buffered saline (PBS), embedded in paraffin and processed for Hematoxylin and Eosin (H&E) staining. Cell morphology was analyzed using light microscopy, which was performed using a Leica LM (Leica Microsystems, Wetzlar, Germany) microscope with 40× objective magnification. The microscope included a CCD camera, which was used to record digital microscopy images. All images were captured at the same resolution of 150 dpi and diameters of cells and nuclei per high-powered field measured through ImageJ software (National Institutes of Health, Maryland, US).

### Clonogenic assays

To supplement the histology in determining viable cell fractions, a clonogenic assay was run for samples of each treatment condition including control. Cells in each sample were counted using a hemocytometer and added to 3 mL of Methocult^®^ (Stemcell Technologies, Vancouver, Canada). Plated in duplicate, colonies were counted after ten days of incubation at 37°C in a 5% CO_2_ environment and compared with the known concentration of inoculants. Proportion of viable cells in the treated populations was recorded for each treatment condition.

### Flow cytometry

For cell-cycle analysis, 48-hour cisplatinum-treated, 48-hour oncotic decay, 36-hour colchicine-treated, or untreated control cells were fixed in 70% (v/v) ethanol (Commercial Alcohols, Brampton, ON) for 1 hour at −20°C. Cells were permeablized by 0.1% (w/v) Triton-X100 (Sigma Aldrich, St. Louis, MO) for 2 minutes at room temperature, and then incubated with propidium iodide/RNase A stain (Molecular Probes, Eugene, OR) for 30 minutes at 37°C in the dark.

For apoptosis-oncosis characterization, 72-hour cisplatinum-treated, 72-hour oncotic decay, or untreated control cells were stained with a viability marker such as propidium iodide (PI), then backgated onto a color dot plot of forward versus side scatter based on PI fluorescence. Cell debris characterized by a very low FSC/SSC and PI negative stain, and were excluded prior to backgating. Doublets were also excluded by forward scatter signal width versus area analysis.

Flow cytometry measurements were performed using a BD LSRII flow cytometer (BD Sciences, San Jose, CA), with 488 nm light exciting PI to emit at a wavelength of 610 nm, captured through the TexasRed bandpass filter. All experiments captured a minimum of 50,000 events per sample.

Cell cycle analysis was performed using FCS Express 5 Multicycle Software (De Novo Software, Glendale, CA).

### Ultrasound data collection and analysis

Ultrasound imaging and RF-data acquisition was performed with a high frequency ultrasound device (UBM) (VS40B, VisualSonics Inc., Toronto, Canada). Two single-element transducers, with center frequencies of 20 MHz and 40 MHz, respectively, were used for the experiments (VisualSonics Inc., Toronto, Canada). A detailed description of the transducer specifications is given in Figure Supplementary S3. Bandwidth values are stated for the −6 dB range relative to the center frequency in the power spectrum. These were obtained by obtaining the peak value of the power spectrum from a quartz disk reference immersed in PBS, from which a Gaussian-fitted function would indicate the frequency range covering −6 dB relative to that maximum value. The ultrasonic device allowed for real time B-mode imaging of the specimen. The sampling frequency of the UBM's built-in analogue to digital converter unit was 500 MHz (input range: ±250 mV; sample resolution: 8 bit).

Centrifuged cell samples were scanned in stainless steel wells [26, 61]. Three samples were scanned for each treatment condition, along with an untreated control sample. A total of 140 RF scan lines were acquired from four different scan planes (35 lines per scan plane) to reduce noise. To obtain uncorrelated and independent signals, scan planes were separated by a distance of at least 250 μm, which is larger than the beam width of the transducer used. The length of the recorded RF-data segments was 3-4 mm, consisting entirely of pellet signal. Acquired data segments were positioned around the focus of the transducer, which was consistently adjusted to 1-2 mm below the pellet surface.

Frequency domain spectral analysis was performed using normalized power spectrum of RF signal with an in-house software in MATLAB (Mathworks, Massachusetts, USA) [21, 34, 64].

The power spectrum analysis is described mathematically by:

$$A_{l}(f,z_{l})=\frac{A_{s}(f,z_{l})}{A_{r}(f,z_{l})}$$ [1]

$$S(f)=\log_{10}\frac{1}{N}{\sum_{l=1}^{N}\left|A_{l}(f,z_{1})\right|}^{2}e^{-4(\alpha_{s}-\alpha_{r})(R+\frac{Dz}{z})}$$ [2]

Where *A_l_* (*f*, *z_l_*) is the amplitude line spectrum of a gated RF line segment (*z_l_*) acquired from the ratio between the fast Fourier transform post-Hanning window gating of an RF line segment of the sample versus a quartz disk calibration reference. This removed any system dependent characteristics from the signal and generated a normalized spectrum [65]. The log power spectrum, *S*(*f*), is computed by averaging the squared magnitudes of each RF line amplitude spectrum laterally across the ROI window, applying a term to compensate for attenuation, and taking the log of the result. For the attenuation compensation term, α_*s*_ and α_*r*_ are the coefficients for the sample and reference data, respectively, R is the axial distance from the transducer to the proximal edge of the ROI window, and Δ_z_ is the axial window length.

Using linear regression analysis, a line of best fit was obtained for the normalized power spectrum within a −6 dB window with respect to the maximum power from the reference spectrum for the transducer. The best fit line was used to determine the mid-band fit (MBF), spectral slope (SS) and the 0-MHz Intercept (SI) parameters [65].

S(f)=SSf+SI [3]

$$\mathrm{MBF}=S(f_{c})$$ [4]

With *f_c_* being the transducer's center frequency.

In addition to the quantitative ultrasound spectral parameters described, backscatter parameters were extracted from ultrasound RF data using an Anderson Fluid-Filled Sphere form factor, modeling the tissue samples as inhomogeneous fluids [29]. The model utilized assumes plane wave incidence, the Born approximation [66], single scattering, and the absence of shear waves [67]. The backscattered signal was modeled as a statistical distribution of scatterers described by average scatterer diameter (ASD) and average acoustic concentration (AAC).

### Statistical analysis

Statistical analysis was performed using GraphPad Prism (Graphpad Software, San Diego, CA). Single-factor, one-tailed ANOVA followed by Tukey post-hoc tests were applied to each combination of conditions, with p<0.05 being considered statistically significant.

## SUPPLEMENTARY FIGURES


