# Supplementary material for: High-frequency ultrasound detection of cell death: Spectral differentiation of different forms of cell death in vitro
Source: Oncoscience. 2016 Sep 12;3(9-10):275–87. doi: 10.18632/oncoscience.319 (PMC5116945; doi:10.18632/oncoscience.319)
Supplement: Supplementary file 1 [file oncoscience-03-275-s001.pdf]

High-frequency ultrasound detection of cell death: Spectral differentiation of different forms of cell death *in vitro*

SUPPLEMENTARY FIGURES

Condition	% Viable
Control	100
Oncosis (72 hrs)	0.0024
Cisplatinum (48 hrs)	0
Heated	0
Colchicine (36 hrs)	0.0055

Supplementary Figure S1: Clonogenic assays were conducted to determine the minimum percentage of viable and affected cells. For all treatments, the vast majority of cells (≥ 99%) were affected, indicating minimal interference from remaining viable cells on the ultrasound signal.

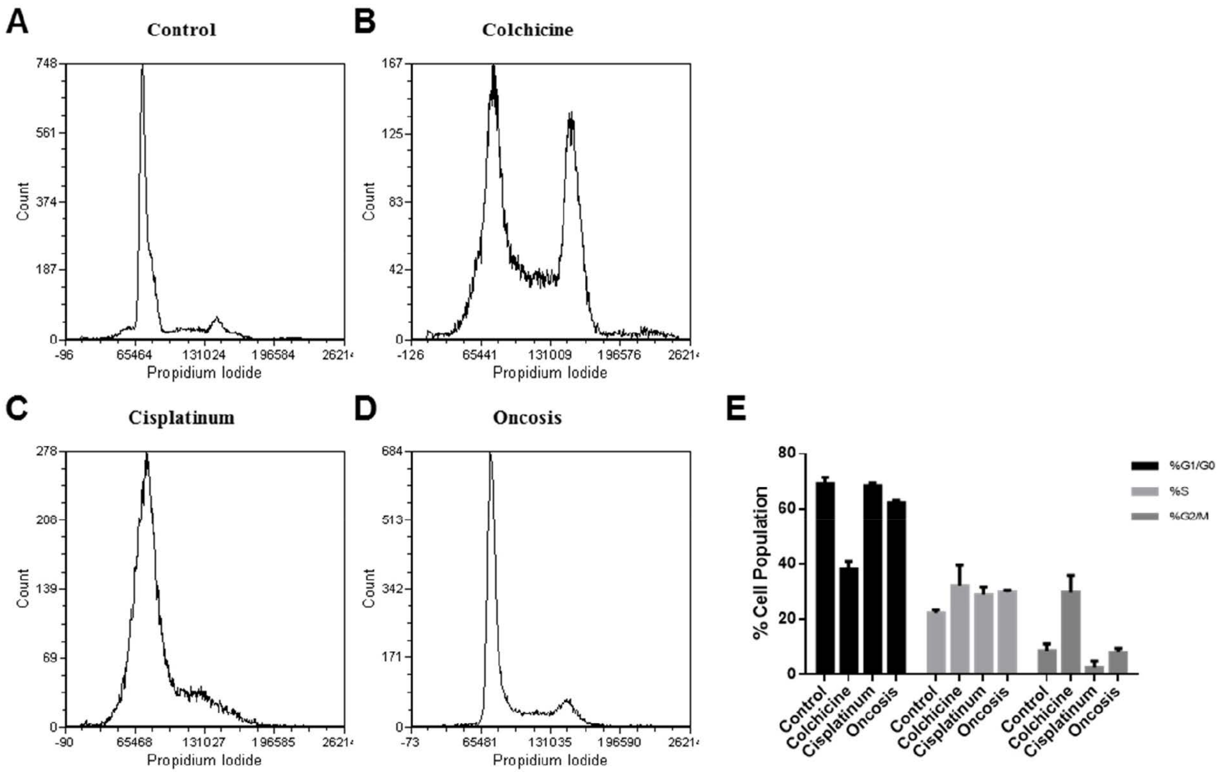

Supplementary Figure S2: Flow cytometry cell cycle representative profiles of A. untreated, B. 36-hour colchicine treatment, C. 48-hour cisplatinum treatment, and D. 48-hour oncosis. E. Quantitative analysis of cell cycle phase percentages of G1/G0, S, and G2/M populations for untreated controls, 36-hour colchicine, 48-hour cisplatinum, and 48-hour oncosis. Error bars represent SD for n=4 per condition.

Transducer	20 MHz	40 MHz
Center frequency (fc)	19.25 MHz	37.5 MHz
- 6 dB Bandwidth (BW)	100%	98%
f-number	2.35	3
Aperture diameter	8.5 mm	3 mm
Depth of focus	3.2 mm	2.5 mm
FWHM (lateral)	247 µm	157 µm
Pulse length	110 ns	50 ns

Supplementary Figure S3: 20 MHz and 40 MHz transducer specifications. Bandwidth values are stated for the -6 dB range relative to the center frequency in the power spectrum.
